# Long‐Lasting Auditory and Vestibular Recovery Following Gene Replacement Therapy in a Novel Usher Syndrome Type 1c Mouse Model

**DOI:** 10.1002/advs.202410063

**Published:** 2025-01-27

**Authors:** Weinan Du, Jun Huang, Aizhen Zhang, Fangfang Zhao, Tianwen Chen, Quinn M. McDermott, Tony Zheng, Haibo Wang, Rongli Zhang, Xiaolin Zhang, Jerome Allison, Hong Zhu, Wu Zhou, Qing Yin Zheng

**Affiliations:** ^1^ Department of Otolaryngology Case Western Reserve University Cleveland OH 44106 USA; ^2^ Department of Otolaryngology‐Head and Neck Surgery University of Mississippi Medical Center Jackson MS 39216 USA; ^3^ Department of Otolaryngology‐Head and Neck Surgery Shandong Provincial ENT Hospital Shandong University Jinan Shandong 250022 China; ^4^ Hearing and Speech Rehabilitation Institute Binzhou Medical University Yantai 264000 China; ^5^ Case Cardiovascular Research Institute Case Western Reserve University School of Medicine Cleveland OH 44106 USA; ^6^ Department of Otolaryngology Binzhou Medical University Hospital Binzhou 256603 China

**Keywords:** USH1C, harmonin, gene replacement therapy, auditory, vestibular, AAV

## Abstract

Usher syndrome type 1C (USH1C) is a genetic disorder caused by mutations in the USH1C gene, which encodes harmonin, a key component of the mechanoelectrical transduction complex in auditory and vestibular hair cells. USH1C leads to deafness and vestibular dysfunction in humans. An *Ush1c* knockout (KO) mouse model displaying these characteristic deficits is generated in our laboratory. To examine gene replacement therapy (GT) in this model, a synthetic adeno‐associated viral vector, *Anc80L65*, driving harmonin expression is administered, to the inner ears of *Ush1c* KO mice at postnatal day 2 (P2). Remarkably, this single treatment significantly improved auditory brainstem response (ABR) thresholds and balance motor function at 1 month post‐injection, with these effects persisting for up to 10 months. At 12 months post‐treatment, the vestibular function is assessed using the vestibular‐ocular reflexes (VOR) and single vestibular afferent recordings. The GT treatment significantly restored both the canal and otolith VORs and increased vestibular afferent spontaneous firing rates and responses to head rotation and translation. These findings provide the first evidence of long‐lasting restoration of both the auditory and vestibular functions by GT in a novel mouse model of Usher syndrome, highlighting the potential of GT for treating deficits associated with USH1C.

## Graphical abstract

This study shows that gene replacement therapy using the *AAV2/Anc80L65* virus can successfully restore hearing and balance in *Ush1c* knockout mice. The treatment leads to lasting improvements in both auditory and vestibular functions, highlighting its potential as a therapeutic approach for genetic hearing loss and vestibular disorders in humans.

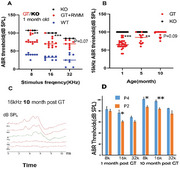

## 1 Introduction

Hearing loss is a global health issue with diverse etiologies, affecting 466 million individuals, or ≈5% of the population.^[^ ^1^ ^]^ A significant risk factor for hearing loss is genetic deficits; as of July 2024, 153 genetic loci have been identified as causes of nonsyndromic hearing loss.^[^ ^2^ ^]^ Usher syndrome (USH) is a group of genetic disorders that cause hearing, balance and vision loss. USH is classified under three distinct subtypes based on the onset and progression of the hearing and vision loss and presence or absence of vestibular symptoms: type I (USH1), type II (USH2), type III (USH3), and type IV.^[^ ^3^ ^]^ USH1 is the most severe form of this disease, characterized by profound congenital bilateral sensorineural hearing loss, vestibular dysfunction, and prepubertal retinitis pigmentosa. The *Ush1c* gene, which encodes the protein harmonin located in the stereocilia and near the ribbon synapse in auditory and vestibular hair cells, was first identified as the cause of the USH1C subtype of USH1 disease.^[^ ^4^ ^]^ We previously generated and characterized a *Ush1c* knockout (KO) mouse model,^[^ ^5^ ^]^ in which the first four exons of the *Ush1c* gene were deleted and replaced with a reporter gene. The homozygous *Ush1c* KO mice exhibit characteristic USH1C phenotypes, including deafness and balance impairments, providing an excellent model for testing therapeutic approaches.^[^ ^5^ ^]^

While cochlear implants restore partial hearing, gene replacement therapy has the potential to provide more complete restoration of auditory sensation. Additionally, inner ear gene replacement therapy treatments can restore balance function which is severely affected in USH1C patients.^[^ ^6^ ^]^ Over the past decade, different modes of therapy have been explored to target the inner ear and restore function in *Ush1c* mouse models. These include the use of small antisense oligonucleotides^[^ ^7^ ^]^ and gene replacement therapy using viral vectors.^[^ ^8^ ^]^ Adeno‐associated viruses (AAVs) are the most promising vectors for gene replacement therapy because they have high infection efficiency, are proven safe,^[^ ^9^ ^]^ are stable, and successfully target sensory hair cells. A synthetic AAV2 vector, Anc80L65, has been shown to target the inner ear with high efficiency and specificity.^[^ ^8^, ^10^ ^]^ Gene replacement therapy using this vector was validated for the treatment of hearing loss associated with a mutation in the *Ush1c* gene, identified in patients of Acadian descent.^[^ ^8^ ^]^ In that work, Pan et al. validated the use of a viral vector encoding harmonin‐b1, one of the isoforms encoded by the *Ush1c* gene. In this study, we examined whether the AAV2‐mediated gene replacement therapy can provide long‐lasting restoration of both auditory and vestibular (canal and otolith) functions in the *Ush1c* KO mice. The vector was injected into the posterior semicircular canal (SCC) or the round window membrane (RWM) of *Ush1c* KO mice at postnatal day 2 (P2) or 4 (P4). Auditory function was assessed using auditory brainstem response (ABR) measurements, and vestibular function was evaluated using vestibular‐ocular reflex (VOR) responses and single vestibular afferent activity in response to head rotation (canal function) and translation (otolith function). Balance motor function was assessed by swimming test and open field observations. We found that gene replacement therapy resulted in substantial improvements in auditory and vestibular functions which were maintained up to 12 months post‐treatment.

## 2 Results

### 2.1 Gene Replacement Therapy Rescues Auditory Function

Ush1c knockout (KO) mice were treated with gene replacement therapy using the AAV2/Anc80L65.CMV.Harmonin‐b1 viral vector (titer: 1.7 × 10^12^ gc mL^−1^), injected at postnatal day 2 (P2) or P4, either through the semicircular canal (SCC) (*n* = 25) or the round window membrane (RWM) (*n* = 25). The injection volumes were either 1 or 1.25 µL.

**Figure 1:**
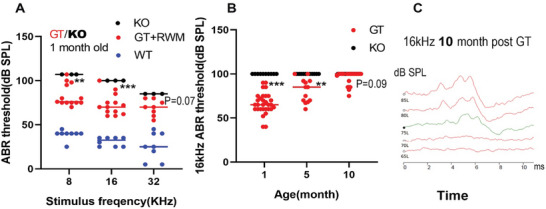
Auditory brainstem response (ABR) of the *Ush1c* KO mice with gene replacement therapy (GT) showed improved hearing. A). ABR thresholds in WT (*n* = 8), GT‐treated (*n* = 13), and untreated (*n* = 3) *Ush1c* KO mice at 1 month post‐treatment via the RWM. B). ABR thresholds in GT treated and untreated *Ush1c* KO mice at 1 (*n* = 30), 5 (*n* = 27), and (*n* = 13) 10 months post‐treatment. C). Representative ABR records of GT‐treated mice at 10 months post‐treatment. Statistics was analyzed by Student's unpaired two‐tailed *t* test. ^*^ *p* < 0.05; ^**^ *p* < 0.01; ^***^ *p* < 0.001.

To evaluate whether gene replacement therapy restored hearing in the Ush1c KO mice, auditory brainstem response (ABR) thresholds were measured at 8, 16, and 32 kHz at 1, 5, and 10 months post‐treatment. We observed improved ABR thresholds in mice treated at P2 or P4. **Figure** **1** shows that at 1 month post‐treatment, ABR thresholds had significantly improved in mice that received RWM injections of the vector. Specifically, average thresholds were 27, 30, and 15 dB SPL for stimuli at 8, 16, and 32 kHz, respectively (Figure 1A). At 10 months post‐treatment, significant recovery was still observed at 16 kHz, with thresholds of 75 dB SPL—an improvement from 52 dB SPL at 1 month post‐treatment (Figure 1B). Considering that the background C57BL/6J strain typically experiences age‐related hearing loss due to the Ahl allele in the CDH23 gene, the preservation of hearing observed at 10 months post‐treatment is promising. Figure 1C displays typical ABR responses of a Ush1c KO mouse at 10 months post‐treatment. Additionally, the abstract graphic D indicates that GT‐treated Ush1c KO mice exhibited significantly improved ABR thresholds with injections given at postnatal day 2 (*n* = 7) compared to those given at postnatal day 4 (*n* = 5), with further significant differences observed as the mice aged; notably, the P4 group became deaf when their hearing was tested at 10 months post‐GT.

**Figure 2:**
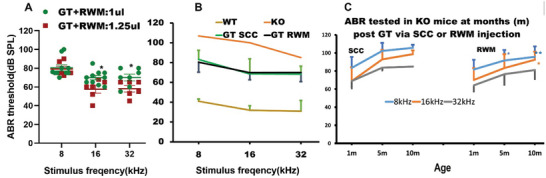
Effects of injection volume and delivery method on gene replacement therapy (GT).A). Comparison of the *Ush1c* KO mice at 1‐month post‐treatment with injections of 1 (*n* = 10) and 1.25 (*n* = 6) µL of the AAV virus via the round window (RWM). B). Comparisons among ABR thresholds at 1‐month post‐treatment via the RWM (*n* = 14), the posterior semicircular canal (SCC) (*n* = 11) and untreated KO (*n* = 3) as well as WT (*n* = 5) mice at P2. C). ABR thresholds at different time points post‐treatment of GT‐treated mice via the RWM (*n* = 12) or the SCC (*n* = 13) at P2. A Student's t‐test was used for significance testing. ^*^ *p* < 0.05; ^**^ *p* < 0.01.

To assess the impact of injection volume on hearing rescue, we compared ABR threshold improvements between the 1 and 1.25 µL conditions (**Figure** **2**A). Gene replacement therapy with 1.25 µL of the virus improved ABR thresholds by 27, 42.5, and 27 dB for 8, 16, and 32 kHz, respectively, indicating substantial restoration of hearing across the cochlea. Although gene replacement therapy using 1.0 µL of the virus also led to significant ABR threshold improvements, the 1.25 µL injection resulted in an additional 12.6 and 12 dB improvement at 16 and 32 kHz, respectively (*P* < 0.05). No significant difference was observed at 8 kHz between the two volume conditions.

### 2.2 Gene Replacement Therapy via the SCC or RWM Leads to Similar Hearing Rescue at 1 Month, with the RWM Showing Better Long‐Term Benefits

We compared hearing improvements between mice treated via the SCC (*n* = 25) and the RWM (*n* = 15) with a 1 µL injection. No significant differences were observed in ABR thresholds at 1–2 months post‐treatment (Figure 2B). However, at 5 months and 10 months post‐treatment, gene replacement therapy via the RWM provided better hearing rescue (Figure 2C) compared to that via the SCC. The specific ABR thresholds observed were as follows: at 5 months, 8 kHz for RWM/SCC was 91.70 ± 11.59 dB/102.33 ± 8.85 dB SPL, and at 10 months, 8 kHz was 95.70 ± 11.50 dB/105.62 ± 3.31 dB SPL, while at 24 kHz it was 92.61 ± 8.85 dB/98.69 ± 4.07 dB SPL.

### 2.3 Gene Replacement Therapy Improves Balance Motor Function in Ush1c Knockout (KO) Mice

**Figure 3:**
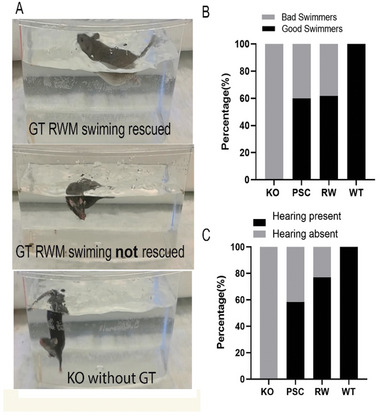
Gene replacement therapy (GT) improves swim capability in *Ush1c* KO mice.A). Mice with improved swimming (“good swimmers”) are able to keep their heads above water (upper panel), while “poor swimmers” and the negative control (*Ush1c* KO) cannot (middle and lower panels).B). Among the GT‐treated mice, 12 out of 20 PSC‐microinjected mice and 13 out of 22 RWM‐microinjected mice were identified as good swimmers.C). Of the 12 and 13 good swimmers from the PSC and RWM injection groups, respectively, 7 and 10 showed improved hearing (as indicated by 16 kHz ABR waves), while the remaining mice did not show hearing improvement. Fisher's exact test was used to compare the difference in hearing improvement, revealing no statistical difference (odds ratio = 0.4464, *p* > 0.05). A Student's t‐test was used for significance testing.

As previously reported in our earlier study,^[^ ^5^ ^]^ Ush1c KO mice exhibited impaired swimming ability (**Figure** **3**A and Video S1, Supporting Information), excessive circling behavior (Video S2, Supporting Information), and hyperactivity. To determine whether gene replacement therapy improved balance motor function in Ush1c KO mice, we conducted swimming and open‐field tests in 20 mice receiving SCC injections and 22 mice receiving RWM injections. Among the SCC‐injected mice, 12 out of 20 demonstrated improved swimming capability (Figure 3B), while 13 out of 22 RWM‐injected mice also showed improvement. Of those with improved swimming capability, 7 out of 12 in the SCC group and 10 out of 13 in the RWM group exhibited improved hearing at all three tested frequencies (Figure 3C). Notably, there was a trend toward a higher success rate in the swimming test for the RWM‐injected mice compared to the SCC‐injected mice.

**Figure 4:**
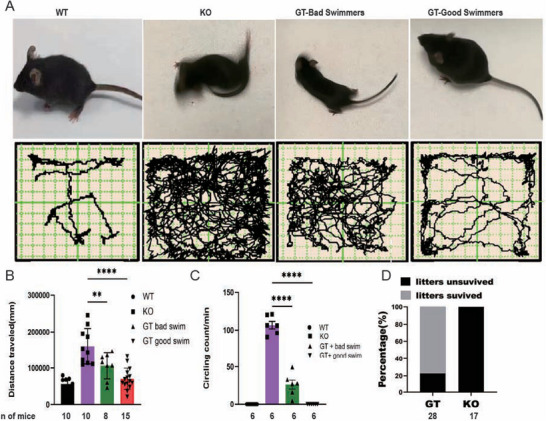
GT reduces circling behavior in *Ush1c* KO mice.A). Representative movement images (upper) and tracks (lower) over the 5 min period for WT mice, untreated KO mice, KO mice treated via the RWM, KO mice treated via the SCC. B). Quantification of travel distance for the four groups. C). Quantification of circling for the four groups of mice. D). Percentage of surviving offspring after GT in KO female mice. Data are presented as mean ± S.D. A Student's *t*‐test was used for significance testing. ^**^ *p* < 0.01; ^****^ *p* < 0.001.

Travel distances and circling behaviors were assessed using the open‐field test. **Figure** **4**A (upper panel) displays representative movement trajectories from each group (for examples of typical circling behavior, see Video S2, Supporting Information). The untreated Ush1c KO mice traveled nearly three times the distance of WT mice (Figure 4A, lower panel, and Figure 4B), whereas gene replacement therapy significantly reduced their travel distances to levels comparable to those of WT mice. Figure 4C compares circling behaviors between the treated and untreated mice. All gene replacement therapy‐treated mice exhibited a significant reduction in circling behavior compared to untreated mice. Treated mice classified as good swimmers did not display circling behavior, while those categorized as poor swimmers had an average of 26.4 circles min^−1^, compared to 106.7 circles min^−1^ in untreated mice.

Interestingly, the effects of gene replacement therapy on hearing and motor function exhibited individual variability. Four mice showed hearing restoration without improvement in swimming ability, while three mice demonstrated improved swimming ability despite no restoration in hearing. When gene replacement therapy was performed on KO mice at P2, 79% (11 out of 14) exhibited a reduction in circling behavior, which is significantly higher than the reduction rate of 33% (4 out of 12) observed when therapy was administered at P4. Notably, all 19 KO mice that displayed no circling behavior at 1 month post‐gene replacement therapy remained free of circling behavior for up to 13 months (data not shown).

### 2.4 Gene Replacement Therapy Restores Vestibulo‐Ocular Reflex (VOR) Responses in Ush1c KO Mice

**Figure 5:**
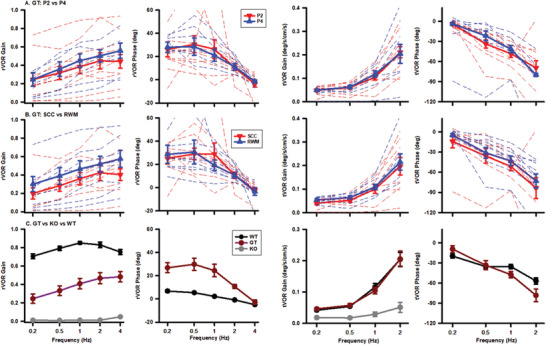
Gene replacement therapy (GT) at P2 or P4 via SCC or RWM restores rVOR and tVOR responses in Ush1c KO mice. A). Gains and phases of the rVOR (left two panels) and tVOR (right two panels) in mice treated at P2 (*n* = 10) or P4 (*n* = 6). The dotted red and blue lines are individual P2 or P4 mice, respectively. Solid red lines and symbols are averages of P2 or P4 mice. B). Gains and phases of the rVOR (left two panels) and tVOR (right two panels) in mice treated via the SCC (*n* = 9) or the RWM (*n* = 7). Dotted red and blue lines represent individual SCC or RWM mice, respectively. Solid red lines and symbols are averages of SCC or RWM mice. C). Averaged gains and phases of the rVOR (left two panels) and tVOR (right two panels) of WT mice (*n* = 6), gene replacement therapy treated mice (*n* = 16) and Ush1c KO mice (*n* = 5).

To investigate whether gene replacement therapy restored vestibular function, we measured VOR responses, which stabilize gaze during head rotation (rVOR) and translation (tVOR). **Figure** **5** illustrates the gains and phases of the rVOR and tVOR in treated and untreated Ush1c KO mice. While untreated mice exhibited near‐zero gains for both sinusoidal head rotations and translations (Figure 5C, grey symbols), the treated mice displayed robust rVOR (*p* < 0.001) and tVOR (*p* < 0.001) responses at all tested frequencies (Figure 5C, dark red symbols), indicating significant recovery of both canal and otolith functions. VOR recoveries were similar between mice receiving AAV2 injections at P2 or P4 (Figure 5A) via the SCC or RWM (Figure 5B), allowing for the combination of results from these groups. While gene replacement therapy led to a significant, though incomplete, recovery of the rVOR, it resulted in nearly full recovery of the tVOR, indicating that the therapeutic effects of gene replacement vary between different end organs.

### 2.5 Gene Replacement Therapy Restores Vestibular Afferent Activities in Ush1c Knockout (KO) Mice

**Figure 6:**
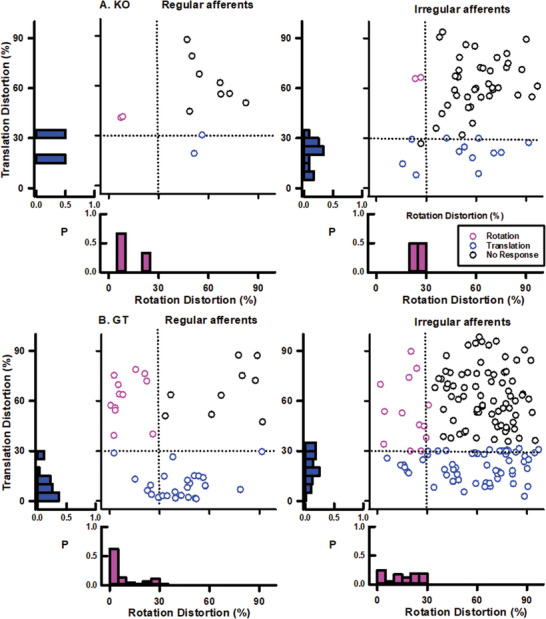
Gene replacement therapy restores vestibular afferent responses to head rotation and translation. Vestibular afferent responses to head rotation and translation in untreated Ush1c KO mice (*n* = (*n* = 8). 5). A) and gene replacement therapy treated mice(*n* = 8). B). Each symbol is a vestibular afferent. Magenta symbols are afferents exhibiting significant responses to head rotation with rotation distortion less than 31% and translation distortion larger than 31%. Blue symbols are afferents exhibiting significant responses to head translation with translation distortion less than 31% and rotation distortion larger than 31%. Black symbols are afferents that do not exhibit significant responses to head rotation and translation tested. In the left panels, vertical dotted lines have a rotation distortion of 31%. In the right panels, the vertical dotted lines are CV^*^ of 0.1, therefore, afferents left of the line are regular afferents, and right of the line are irregular afferents. The bars are histograms of the corresponding measurements (rotation distortion, translation distortion, and CV^*^). For more details, see Tables 1 and 2.

To further assess the effects of gene replacement therapy on the restoration of vestibular function, we performed single‐unit recordings of vestibular afferents in 5 untreated and 8 treated Ush1c KO mice at 12 months post‐treatment. A total of 108 vestibular afferents were recorded from untreated mice and 311 from treated mice. Afferents were classified as head‐rotation (canal) units, head‐translation (otolith) units, or non‐responsive units based on their responses to horizontal head rotation and translation (**Figure** **6**, left panels). They were further classified as regular or irregular units based on their coefficient of variation (CV^*^) (Figure 6, right panels). Gene replacement therapy significantly increased the number of afferents responsive to both head rotation and translation (Chi‐square = 16.3, P = 0.001) (**Table** **1**). In untreated Ush1c KO mice, only 18.5% (20 out of 108) of recorded vestibular afferents responded to head rotation (3 out of 108, 3.7%) or translation (17 out of 108, 15.7%). In contrast, 47.9% (149 out of 311) of recorded vestibular afferents in treated mice responded to either head rotation (63 out of 311, 20.2%) or translation (86 out of 311, 27.7%). Additionally, gene replacement therapy significantly increased the number of regular vestibular afferents (Chi‐square = 6.7, P = 0.01). In untreated mice, 11.1% (12 out of 108) of recorded vestibular afferents were classified as regular units, comprising 1 (0.9%) canal unit and 2 (1.9%) otolith units. However, in treated mice, 30.2% (94 out of 311) of recorded vestibular afferents were regular units, with 40 (12.9%) canal units and 27 (8.9%) otolith units.

**Table 1:** Effects of gene replacement therapy (GT) on vestibular afferent responses to head rotation (canal) and translation (otolith).

Mice	All afferents			Canal afferents			Otolith afferents		
	Total	Regular	Irregular	Total	Regular	Irregular	Total	Regular	Irregular
*Ush1c* KO (*n* = 5)	108	12	96	3	1	2	17	2	15
GT treated (*n* = 8)	311	94	217	63	40	23	86	27	59

**Figure 7:**
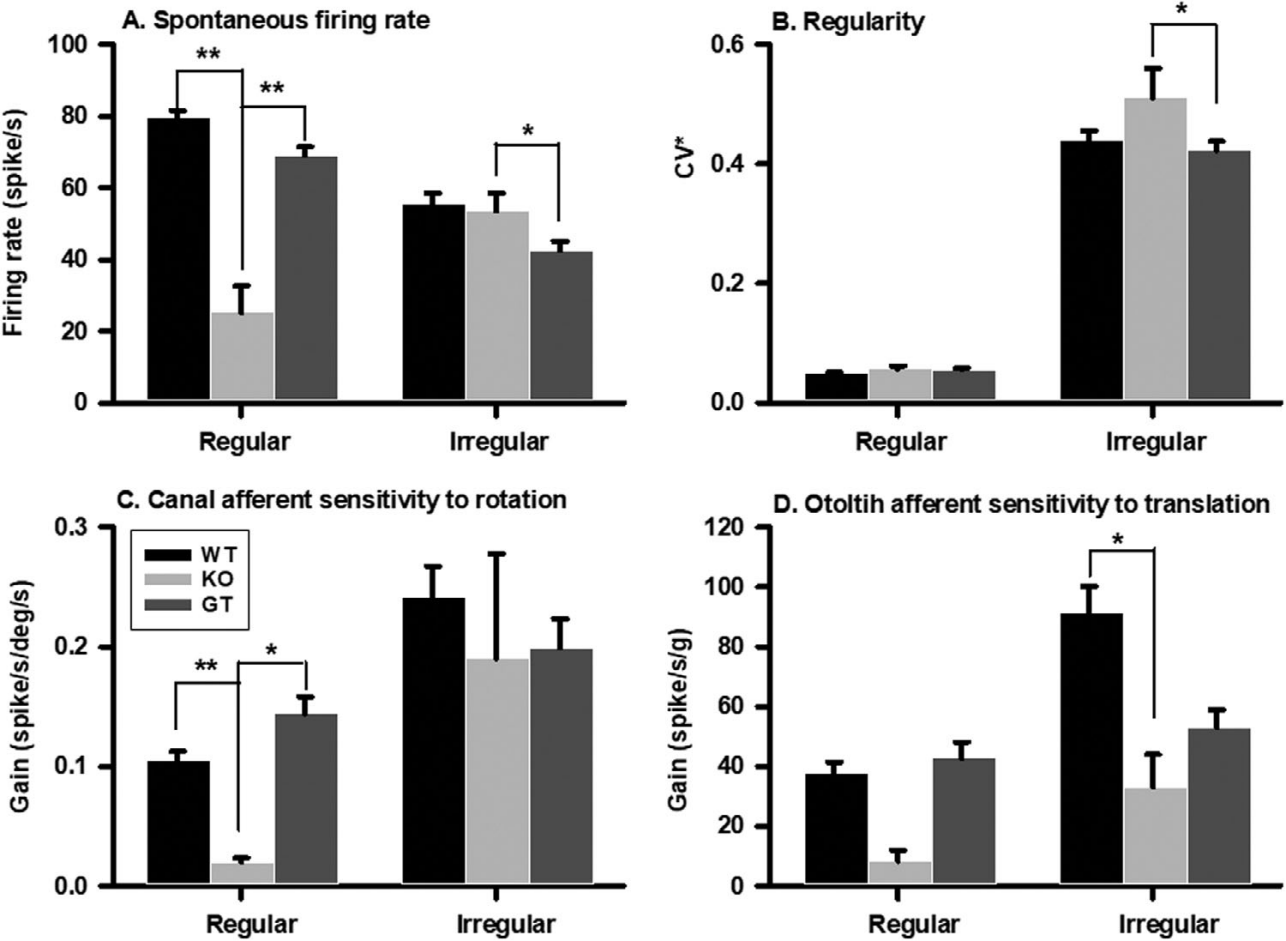
Gene Replacement Therapy (GT) Restores Vestibular Afferent Spontaneous Firing Rate A), Regularity B), and Sensitivity to Head Rotation C) and Translation D). Panel A presents the spontaneous firing rate of vestibular afferents, while Panel B illustrates the regularity of firing. Panels C and D depict the sensitivity of afferents to head rotation and translation, respectively. For additional details, please refer to Table 2. Asterisks indicate statistical significance as determined by *t*‐tests: ^*^, *P* < 0.05; ^**^, *P* < 0.001.

**Figure** **7** illustrates the effects of gene replacement therapy on the spontaneous firing rate, regularity, and sensitivity of vestibular afferents to head rotation and translation (for additional details, see **Table** **2**). Gene replacement therapy significantly increased the spontaneous firing rates of regular afferents (Treated: 69.48 ± 2.63 spike/s, *n* = 94; Untreated: 26.64 ± 8.46 spike s^−1^, *n* = 12; *P* < 0.001), while it slightly decreased the spontaneous firing rates of irregular afferents (Treated: 46.15 ± 2.92 spike s^−1^, *n* = 217; Untreated: 53.65 ± 5.21 spike s^−1^, *n* = 96; P = 0.0337). Gene replacement therapy did not significantly change the CV^*^ of the regular afferents (Treated: 0.06 ± 0.002, *n* = 94; Untreated: 0.06 ± 0.005, *n* = 12), but it slightly decreased the CV^*^ of the irregular afferents (Treated: 0.45 ± 0.02, *n* = 217; Untreated: 0.53 ± 0.055, *n* = 96; P = 0.0196). Additionally, gene replacement therapy increased afferent sensitivity to head rotation and translation. For canal afferents, regular unit sensitivity to head rotation increased from 0.011 spike/s/deg/s (*n* = 1) to 0.15 spike/s/deg/s (*n* = 40), while irregular unit sensitivity to head rotation increased from 0.07 spike/s/deg/s (*n* = 2) to 0.22 spike/s/deg/s (*n* = 25) (P = 0.222). For otolith afferents, regular unit sensitivity to head translation increased from 8.20 spike/s/g (*n* = 2) to 42.69 spike/s/g (*n* = 27) (P = 0.0769), while irregular unit sensitivity to head translation increased from 24.23 spike/s/deg/s (*n* = 12) to 54.42 spike/s/deg/s (*n* = 57) (P = 0.0523). These results were consistent with the observed recoveries of the rVOR and tVOR responses in the treated mice.

**Table 2:** Summary of vestibular afferent spontaneous firing rates (FR), regularity (CV^*^) and sensitivity to rotation (Rot‐Gain) and translation (Tran‐Gain) in GT treated and untreated mice (Mean ± SEM).

	Type	FR[spike s^−1^]	CV^*^	Rot‐Gain [spike/s/deg/s]	Tran‐Gain[spike/s/g]
*Ush1c* KO (N = 5)	All (108)	50.05±4.73	0.46±0.050		
	Regular (12)	26.64±8.46	0.06±0.005		
	Irregular (96)	53.65±5.21	0.53±0.055		
	Canal (3)	36.63±17.87	0.23±0.074	0.055±0.029	
	Regular (1)	9.23±0.00	0.05±0.000	0.018±0.005	
	Irregular (2)	45.76±21.73	0.28±0.063	0.070±0.036	
	Otolith (17)	39.48±12.53	0.26±0.049		21.31±7.13
	Regular (2)	10.40±5.65	0.07±0.003		8.20±3.64
	Irregular (15)	45.30±14.39	0.30±0.051		24.23±8.45
GT treated (N = 8)	All (311)	53.20±2.27	0.33±0.016		
	Regular (94)	69.48±2.63	0.06±0.002		
	Irregular (217)	46.15±2.92	0.45±0.017		
	Canal (63)	63.94±4.28	0.17±0.025	0.18±0.020	
	Regular (40)	72.34±3.60	0.05±0.002	0.15±0.015	
	Irregular (23)	50.51±9.00	0.37±0.044	0.22±0.046	
	Otolith (86)	55.17±4.71	0.32±0.031		50.65±4.68
	Regular (27)	72.05±5.18	0.06±0.004		42.69±5.45
	Irregular (59)	43.66±5.26	0.44±0.036		54.42±6.36

### 2.6 Immunofluorescence Reveals High Levels of Harmonin in Both Auditory and Vestibular End Organs

**Figure 8:**
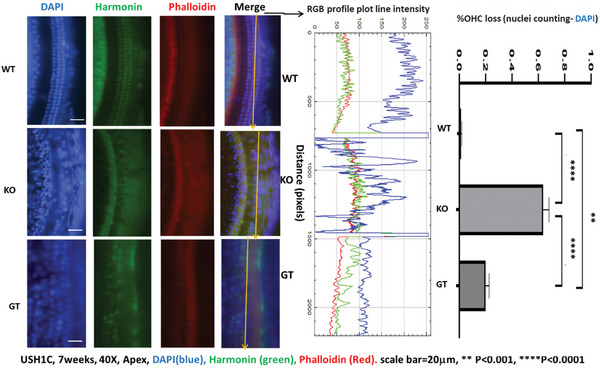
Increased Harmonin Expression and Reduced Outer Hair Cell (OHC) Loss in Gene Replacement Therapy‐Treated (GT) Ush1c Knockout (KO) Mice Compared to Untreated KO Mice. The left panel shows apex turns of cochleae stained with an anti‐Harmonin antibody (green) in untreated Ush1c wild‐type (WT) mice, untreated Ush1c KO mice, and Ush1c KO mice treated with gene therapy (GT). The RGB profile plots illustrate 2D graphs of the raw fluorescence intensities corresponding to the yellow arrows drawn in the merged panels. The *Y*‐axis represents distance (in pixels) along the lines, while the *X*‐axis indicates the RGB intensity (red, gray, blue). Harmonin (green) signals were expressed unevenly, with higher peaks (specific staining) and lower troughs indicative of background staining in GT and WT mice. Notably, the DAPI staining in the far left panel was used for total OHC counting, with results presented in the far right panel (*n* = 3 mice per group). The peaks in the blue line profile plots serve as a reference for OHC counts, confirming that a significant number of outer hair cells (OHCs) were preserved following the single GT treatment compared to untreated KO mice.

**Figure 9:**
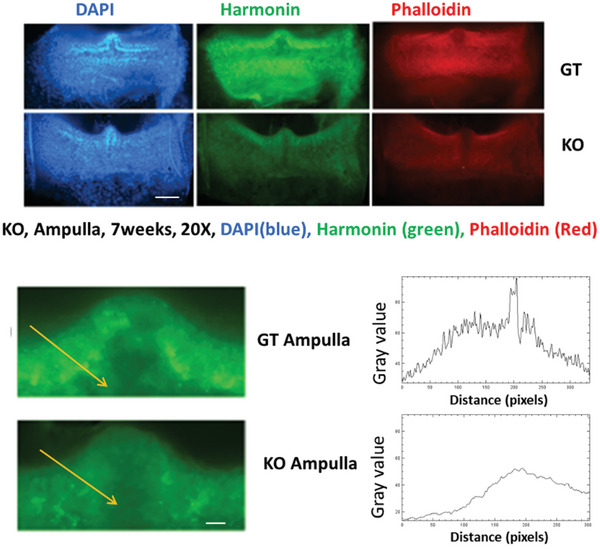
Increased Harmonin Expression in Gene Replacement Therapy‐Treated (GT) Ush1c Knockout (KO) Mice Compared to Untreated KO Mice. The top panel presents a 20x low magnification image showing robust staining of Harmonin expression (green) along the line of hair cells in the ampulla, one of the vestibular organs. The bottom‐left panel displays ampullae stained with an anti‐Harmonin antibody (green) in Ush1c KO mice treated with gene therapy (GT) and untreated Ush1c KO mice. In the bottom‐right panel, the line analysis plots illustrate 2D graphs of the raw fluorescence intensities corresponding to the yellow arrows drawn in the left panels. The *X*‐axis represents the distance (in pixels) along the lines, while the *Y*‐axis indicates the intensity of the gray background. Harmonin (green) signals show higher intensities in cristae, including the hair cell regions of GT‐treated mice compared to untreated KO mice.

Ush1c knockout (KO) mice, both treated and untreated, as well as wild‐type (WT) mice, were sacrificed at 1–2 months of age for immunofluorescence analysis of the cochlear basilar membranes and ampullae. Consistent with our Auditory Brainstem Response (ABR) findings, the immunofluorescence results demonstrated that Harmonin protein is expressed in both inner and outer hair cells, with an outer hair cell loss rate of 20% ± 2.6% in treated Ush1c KO mice. In contrast, untreated Ush1c KO mice exhibited significant hair cell loss (63.7% ± 4.5%), with no detectable Harmonin expression (**Figure** **8**). Notably, Harmonin expression in the treated samples differed from that in the WT cochlear samples, aligning with the limited hearing recovery observed in the treated subjects. Additionally, **Figure** **9** illustrates elevated Harmonin expression in the ampullae of treated mice compared to untreated Ush1c KO mice (Figure 9 – lower right).

### 2.7 Variability in Therapeutic Outcomes Among Individual Mice and Across Different Tests

To investigate the variability in therapeutic outcomes among individual mice and across different tests, we have included the raw data in (Table S1, Supporting Information) for the longitudinal study. In addition to the detailed analysis provided above, we observed that injections into the posterior semicircular canal (SCC) resulted in longer‐lasting vestibular protection effects. For instance, mice #2, 3, and 4 exhibited no circling behavior at 16 months of age, although no protection for hearing was noted. In contrast, mice #204 and #205, which received round window injections, displayed measurable hearing at 10.5 months of age but exhibited vestibular dysfunction characterized by circling behavior, despite one of the mice demonstrating normal swimming ability. This discrepancy may be attributed to the sharper angling of the microinjection glass needle tips toward the cochlea, potentially influencing the distribution of the therapeutic agent. However, we did note an increase in body weight as the mice aged. Investigating the implications of this weight gain will be part of a separate study, as our primary focus in this research is on the inner ear.

## 3 Discussion

In this study, we utilized the AAV2/*Anc80L65* virus (1.0 or 1.25 µL) to deliver a functional harmonin transcript‐b1 into the inner ears of Ush1c knockout (KO) mice at postnatal days 2 (P2) or 4 (P4), employing either the semicircular canal (SCC) or round window membrane (RWM) delivery methods. We observed significant recovery in both auditory and vestibular functions at 1 month post‐treatment, with sustained improvements observed for up to 12 months, underscoring the potential of AAV gene replacement therapy for achieving long‐lasting restoration of genetic hearing loss and vestibular deficits in humans.

Recent studies have explored various serotypes of AAV to achieve targeted transduction of inner ear structures.^[^ ^10^, ^11^ ^]^ AAV2‐based vectors, in particular, have shown notable effectiveness in delivering therapeutic genes to auditory hair cells.^[^ ^11a^ ^]^ Similarly, AAV8 and AAV9 vectors have demonstrated promising results in transducing cochlear cells and restoring auditory function.^[^ ^11i^ ^]^ Notably, the enhanced transduction efficiency of AAV2/Anc80 vectors compared to traditional AAV2 has been well documented, facilitating gene expression even in the challenging inner ear environment. Research by other investigators has also demonstrated that AAV2/Anc80 vectors can effectively deliver genes to both the auditory and vestibular systems, showcasing their dual applicability in treating conditions such as vestibular dysfunction alongside hearing loss.^[^ ^11a,i^ ^]^

The AAV2/Anc80L65 vector utilized in our research is particularly noteworthy due to its improved transduction efficiency and broader tropism for both auditory and vestibular structures, as characterized by Geleoc's group.^[^ ^8^ ^]^ This enhancement allows for a more effective gene therapy approach targeting both auditory and vestibular dysfunctions. The specific vector employed in our study, namely AAV2/Anc80L65.CMV.Harmonin‐b1, highlights the potential for improved outcomes by specifically targeting the pathways involved in hearing loss and recovery, thereby establishing a benchmark for future therapeutic strategies in hearing restoration.

Consistent with the findings of Pan et al. ^[^ ^8^ ^]^ our results demonstrate that gene replacement therapy effectively restored both hearing and balance motor functions in Ush1c KO mice. At the 1.25 µL dose, improvements in auditory brainstem response (ABR) thresholds ranged from 15 to 40 dB. The 1.0 µL dose still produced significant ABR threshold improvements; however, the gains were ≈12 dB lower at 16 and 32 kHz compared to the 1.25 µL dose. Notably, the improvements at 8 kHz were comparable between both doses, indicating that the increased volume enhanced AAV transfection in the middle and basal cochlear regions, while not significantly improving transfection in the apex region (8 kHz).

Gene replacement therapy significantly improved swimming capability and reduced circling behavior in Ush1c knockout (KO) mice. Interestingly, while gene replacement therapy administered at postnatal days 2 (P2) or 4 (P4) demonstrated hearing improvements, treatment at P2 resulted in greater therapeutic effects in reducing circling behavior compared to P4. This finding suggests that P2 may represent a critical window for rescuing vestibular‐motor function using this approach. Conversely, treatment at P4 provided sustained hearing rescue, lasting up to 10 months post‐treatment, compared to the treatment at P2.

In conjunction with the partial dissociation observed among various vestibular measurements and motor functions, we speculate that the natural expression of the Ush1c gene, which includes several known and unknown splice variants in different cochlear and vestibular hair cells, occurs at varying locations and times. This suggests that our gene replacement therapy can only partially mimic natural gene expression. Nevertheless, the downstream effects involving sensory hair cells, synapses, nerve pathways, central nervous system reflexes, and critical learning capabilities may contribute to the observed phenotypic discrepancies, presenting both challenges and avenues for further exploration in our understanding of these mechanisms.

In addition to the effects of gene replacement therapy on hearing and general balance motor behavior, we utilized vestibulo‐ocular reflex (VOR) measurements and single vestibular afferent recordings to further evaluate the effects of gene therapy (GT) on vestibular function. To our knowledge, this is the first study assessing the effects of GT on vestibular function in USH1C using responses of eye movements and vestibular afferents during head rotation and translation. This advancement is crucial for GT studies focused on vestibular function, as vestibular processes are mediated by multiple subsystems involving three populations of vestibular afferents (regular, dimorphic, and calyx) innervating two types of hair cells (Type I and Type II) across five vestibular end organs (three cristae and two maculae).^[^ ^12^ ^]^

It is important to consider the specificity and sensitivity of the assay chosen to test vestibular function. For instance, the reflexive vestibulo‐ocular reflex (rVOR) effectively assesses canal function but is not suitable for evaluating otolith function, which should instead be tested using the translational vestibulo‐ocular reflex (tVOR). Popular tests like the open field, swimming, and rotarod primarily assess gross motor functions related to balance control but lack specificity and sensitivity to identify vestibular deficits.^[^ ^13^ ^]^ In contrast, in vivo single vestibular afferent recordings allow us to characterize a large population of vestibular afferents in terms of their spontaneous discharge rates, regularity, and responses to head rotation and translation. Although technically challenging, this assay is both specific and sensitive to vestibular deficits, and is considered the “Gold Standard” for assessing vestibular peripheral function in animal models. This is because peripheral vestibular afferents transmit the outputs of mechanoelectrical transduction (MET) processes to the central nervous system, which integrates head movement signals for motor control and cognition.

Although Ush1c knockout (KO) mice are known to have balance disorders, it is particularly noteworthy that they exhibited near‐zero gains in the reflexive vestibulo‐ocular reflex (rVOR) and translational vestibulo‐ocular reflex (tVOR), indicating that head rotation and translation evoked minimal modulation in the vestibular afferents that provide inputs to the VOR pathways. Given that VOR responses are primarily driven by the activity of regular afferents,^[^ ^12^ ^]^ it is likely that these regular afferents are severely compromised in untreated Ush1c KO mice. Results from single afferent recordings support this prediction. Figure 6 shows a substantial reduction in the number of regular afferents responsive to head rotation and translation in untreated Ush1c KO mice. The few regular afferents that were recorded exhibited low spontaneous firing rates and reduced sensitivities to head motion. Furthermore, single afferent recording results revealed significant deficits in irregular afferents. Specifically, 37.8% (82/217) of irregular afferents were responsive to head movement in the GT‐treated Ush1c KO mice, compared to only 17.7% (17/96) in untreated mice (Table 2). This suggests that the loss of Harmonin proteins impairs both Type I and Type II hair cells' ability to convert head movement signals into electrical signals.

Gene replacement therapy increased the proportion of regular afferents responsive to head rotation and translation, as well as their spontaneous firing rates and sensitivities (Figures 6, 7). It also enhanced the responsiveness of irregular afferents to head movement. These results indicate that gene replacement therapy effectively corrects the Harmonin gene, facilitating the production of functional Harmonin proteins that restore both Type I and Type II vestibular hair cells in the canals and otoliths. Notably, while GT resulted in a nearly complete recovery of the tVOR, recovery in the rVOR was ≈50%. Given that VOR responses are mediated by both peripheral and central components, differences in either may contribute to the observed disparities in GT effects on rVOR and tVOR. As shown in Table 2, GT induced similar recoveries of spontaneous firing rates and sensitivity to head movement in both canal and otolith afferents. Therefore, it is possible that the central mechanisms responsible for integrating the improved peripheral inputs to generate VOR responses differ between the rVOR and tVOR. Future studies are needed to investigate this important issue further.

Another important issue that warrants further investigation is the finding that 17.7% of vestibular afferents responded to head rotation and translation in untreated Ush1c knockout (KO) mice (Figure 6). In current mechanotransduction (MET) models of hair cells,^[^ ^14^ ^]^ Harmonin proteins play a critical role in the opening and closing of MET channels through tip links. Given the absence of Harmonin proteins in the hair cell stereocilia of Ush1c KO mice, we did not expect to record any vestibular afferent responses during head movements. While it is premature to speculate on the mechanisms underlying these vestibular afferent responses in the absence of Harmonin, these results raise an intriguing question about the existence of MET complexes in vestibular hair cells, particularly in the otoliths, that may operate independently of the Harmonin‐tip link components. Further studies are needed to explore this compelling hypothesis.

While the restorative effects on hearing appear to diminish over time, the recovery of vestibular function, as measured by VOR responses, remains stable even at 12 months post‐treatment. Notably, the tVOR responses at this time point are comparable to those of wild‐type (WT) mice. In contrast to the restoration of circling behavior, which is more effective when therapy is administered at P2 rather than P4, vestibular recovery is similar regardless of whether therapy is administered at P2 or P4. This suggests that gene replacement therapy may have different effects on the sub‐motor systems underlying these behavioral tasks, warranting further investigation.

In this study, we directly compared the efficacy of gene replacement therapy via the semicircular canal (SCC) and the round window membrane (RWM). The RWM, a semipermeable membrane that separates the middle and inner ear, is commonly used for hearing correction through gene therapy, while SCC injection has also been employed and is suggested to be more effective for targeting vestibular hair cells. Our results indicate that gene replacement therapy via both the RWM and SCC yields similar improvements in auditory brainstem response (ABR) thresholds and vestibulo‐ocular reflex (VOR) responses. Given the convenient access to the RWM, our findings position it as a viable target site for clinical gene replacement therapy aimed at vestibular dysfunction. The primary advantage of RWM delivery lies in its long‐term efficacy for hearing restoration, whereas the benefits of SCC delivery are primarily associated with enhanced long‐term vestibular function. This distinction may stem from the distance and diffusion characteristics of the adeno‐associated virus (AAV) carrying the therapeutic gene.

In addition to restoring auditory and vestibular functions, as well as general balance and motor coordination in Ush1c knockout (KO) mice, gene replacement therapy also reinstated the ability of female mice to effectively nurse their offspring. Rather than addressing a presumed issue with milk production, the enhanced feeding capability is likely attributable to the cessation of circling behavior, which enables the mother mice to nurse their pups more efficiently.

Concerning potential off‐target effects of gene therapy and the long‐term safety profile, we did not observe any significant adverse effects, such as tumor formation or weight loss, which is promising for future clinical applications. We noted weight gain with aging in a significant number of GT mice, although this was not observed in all subjects. Therefore, considerations regarding off‐target effects, gene deterioration, and immune clearance of the delivered gene should be included when evaluating long‐term outcomes for human subjects, especially given that humans typically have a significantly longer lifespan than mice.

The methodologies developed in this study demonstrate significant potential for application to other genetic diseases characterized by similar pathophysiological processes. The AAV2/Anc80L65 gene replacement therapy can potentially be adapted for conditions such as Usher syndrome, which is caused by mutations in genes like USH1C, leading to hearing loss, vestibular dysfunction, and retinitis pigmentosa. Additionally, the targeted delivery methods via the semicircular canal (SCC) and round window membrane (RWM) may be applicable to other disorders associated with auditory and vestibular defects, including those linked to ototoxicity.

Furthermore, AAV vectors can be engineered for broader therapeutic applications, extending to conditions such as retinitis pigmentosa and various inherited neuropathies. Our approach, which demonstrates long‐lasting gene expression in the Ush1c KO mouse model, suggests that similar strategies could enhance gene therapies for a range of genetic disorders, ultimately providing substantial benefits to affected patients.

## 4 Conclusion and Future Studies

In summary, our study demonstrates that inner ear gene replacement therapy using the harmonin‐containing AAV2/*Anc80L65* virus successfully achieves long‐lasting restoration of auditory and vestibular functions, including both canal and otolith activities. Future research should focus on several key areas: investigating the efficacy of AAV viruses containing other harmonin isoform transcripts, evaluating the effects of gene replacement therapy administered at various postnatal time points, elucidating the mechanisms underlying the rescue of hearing and vestibular function, and exploring the mechanotransduction (MET) processes that may involve non‐harmonin mechanisms.

## 5 Experimental Section

### Animals

Ush1c knockout (KO) mice, previously generated in our laboratory, along with genetically matched C57BL/6J mice, were maintained at the animal resource facility of Case Western Reserve University. All procedures were conducted in accordance with NIH guidelines for the care and use of laboratory animals. The experimental protocols (protocol #s 2014‐0155, R01DC015111, and R01DC008853) received approval from the Institutional Animal Care and Use Committees at both Case Western Reserve University and the University of Mississippi Medical Center (protocol #0932E and #0932F). Both male and female mice were utilized in comparable numbers across all studies.

### Genotyping

Mice were genotyped using a tail clip method as previously described. Briefly, tissue samples were solubilized in 1 m NaOH at 94 °C for 10 min, followed by the addition of 1 m Tris/HCl (pH 8.0) and subsequent neutralization with 2 m HCl. The supernatant obtained after centrifugation was used as a template for PCR, which was performed as outlined in reference,^[^ ^5^ ^]^ and the resulting amplicons were analyzed using 1% agarose gel electrophoresis (Figure S1, Supporting Information).

### Viral Vectors

The following viral vector was generously donated by Dr. Gwenaelle S.G. Geleoc's group. Detailed characterization and expression profiles in mouse inner ears have been previously described.^[^ ^10^ ^]^ The following titer was utilized in this study:

1. AAV2/*Anc80L65*.CMV.Harmonin‐b1: 1.7 × 10^12^ gc mL^−1^.

The viruses were aliquoted and stored at ‐80 °C until thawed just prior to surgery.

### RWM and SCC Injection

Following genotyping and confirmation as Ush1c knockout (KO) mice, pups aged P2–P4 received injections via either the round window membrane (RWM) or the posterior semicircular canal (SCC). The pups were anesthetized using hypothermia on ice for 5–10 min prior to viral injection. Trypan blue was utilized to fill the glass pipette tips for visible microinjection practice into the target locations, followed by practice injections with phosphate‐buffered saline (PBS). The virus was thawed on ice immediately before the experiment, and the injection rate was set to 10 nL s^−1^ using a SMARTouch controller (World Precision Instruments, Sarasota, FL). The total injection volume was either 1 or 1.25 µL, with the volume of injected materials regulated at ≈0.02 µL min^−1^ for a duration of 10 min. The incision was sealed using tissue adhesive (3M, St. Paul, MN), and the pups were placed on a heating pad to maintain warmth after injection before being returned to their mother once they regained consciousness.

### ABRs

Mice aged 1–10 months were weighed and tested for auditory brainstem response (ABR) thresholds using a computer‐aided evoked potential system (Intelligent Hearing Systems, Miami, FL, USA).^[^ ^15^ ^]^ The mice were anesthetized and kept warm on a heating pad. Subdermal needle electrodes were inserted near the left ear, and responses to 8, 16, and 32 kHz tone burst stimuli were recorded. Both Ush1c KO and wild‐type (WT) mice were included in each measurement to ensure system functionality. A total of 25 mice were injected via the PSC and 25 via the RWM. Hearing rescue was defined as a measurable ABR value for stimuli at 8, 16, or 32 kHz.

### Swim Test for Balance Motor Assessment

Ush1c wild‐type (WT) mice served as normal controls, while untreated Ush1c knockout (KO) mice acted as negative controls. Injected Ush1c KO mice were tested in a water tank (20 × 15 × 15 cm^3^) at 4–7 weeks of age. All tests were videotaped. Mice that could keep their heads above water and swim forward were classified as normal, whereas untreated Ush1c KO mice could not maintain head elevation or swim normally.

### Circling Behavior Assessment

Mice were placed in an open box (40 × 30 cm^2^) and videotaped for 5 min while awake. Circling behavior was evaluated by reviewing the footage. Four groups of mice, each consisting of six individuals, were included: untreated Ush1c KO mice, WT mice, microinjected Ush1c KO mice with good swim test results, and microinjected Ush1c KO mice with poor swim test results.

### Open‐Field Experiment

Mice were placed in a BASi Force Plate Actimeter and allowed to explore freely for up to 5 min. The Actimeter, housed in a sound‐attenuating cabinet (kindly provided by Dr. Alberto Costa),^[^ ^16^ ^]^ electronically tracked mouse movement, and all experiments were video recorded for archival purposes. The experiment involved 10 C57BL/6J mice, 10 Ush1c KO mice, 16 gene replacement therapy‐treated Ush1c KO good swimmers, and 9 poor swimmers (age‐matched). The distance traveled by mice aged 1–13 months was analyzed by compiling data from all recorded frames.

### Vestibulo‐Ocular Reflex (VOR)

VOR tests were performed on C57BL/6J wild‐type (WT) mice, Ush1c knockout (KO) mice, and gene replacement therapy‐treated Ush1c KO mice, as previously described.^[^ ^17^ ^]^ Briefly, a head holder was implanted in each mouse, and eye movements were measured using an ETS‐200S (ISCAN, Burlington, MA) mounted on a vestibular stimulator (Neurokinetic, Pittsburgh, PA). Rotations were delivered at frequencies of 0.2, 0.5, 1, 2, and 4 Hz, while translations were delivered at 0.2, 0.5, 1, and 2 Hz. Eye and head signals were acquired using a CED system, and VOR responses were analyzed using Spike2, MATLAB, and SigmaPlot. The gains and phases of the VOR were calculated by performing a Fast Fourier Transform (FFT) on the averaged eye velocity signal and the head rotation or translation velocity signal.

### Vestibular Afferent Recording

Vestibular afferent recordings were conducted under ketamine/xylazine anesthesia on WT mice, Ush1c KO mice, and gene replacement therapy‐treated Ush1c KO mice, following previously established methods.^[^ ^17^, ^18^ ^]^ Briefly, the animal's head was stabilized on a stereotaxic frame using a head holder, and core body temperature was maintained at 36–37 °C. A craniotomy was performed to access the vestibular nerve with a microelectrode. For each spontaneously active nerve fiber, its spontaneous activity and responses were recorded to head rotations and translations using the CED system at 20 kHz with 16‐bit resolution and a temporal accuracy of 0.01 ms. The regularity of spontaneous discharge in vestibular afferents was assessed by calculating the normalized coefficient of variation of interspike intervals (CV^*^), classifying afferents as regular (CV^*^ ≤ 0.1) or irregular (CV^*^ > 0.1).^[^ ^19^ ^]^ Gains and phases relative to head rotation velocity and translation acceleration were quantified using FFT analysis at 1 Hz. In addition to traditional gain metrics, a distortion metric defined as 1 – (Amplitude of the fundamental response)/(Square root of the sum of the squared amplitudes of the first 10 harmonics) was computed, providing a statistical evaluation of head movement signals. This distortion metric was analogous to “stimulus‐response coherence,” which is used for the reliable analysis of weak responses.^[^ ^20^ ^]^

## Conflict of Interest

The authors declare no conflict of interest.

## Supporting information



Supporting Information

Supplemental Table 1

Supplemental Video 1

Supplemental Video 2

Supplemental Video 3

Supplemental Video 4

Supplemental Video 5

## Data Availability

The data that support the findings of this study are available from the corresponding author upon reasonable request.
