# Supplementary material for: Long‐Lasting Auditory and Vestibular Recovery Following Gene Replacement Therapy in a Novel Usher Syndrome Type 1c Mouse Model
Source: Adv Sci (Weinh). 2025 Jan 27;12(29):2410063. doi: 10.1002/advs.202410063 (PMC12362801; doi:10.1002/advs.202410063)
Supplement: Supplementary file 1 — Supporting Information [file ADVS-12-2410063-s002.docx]

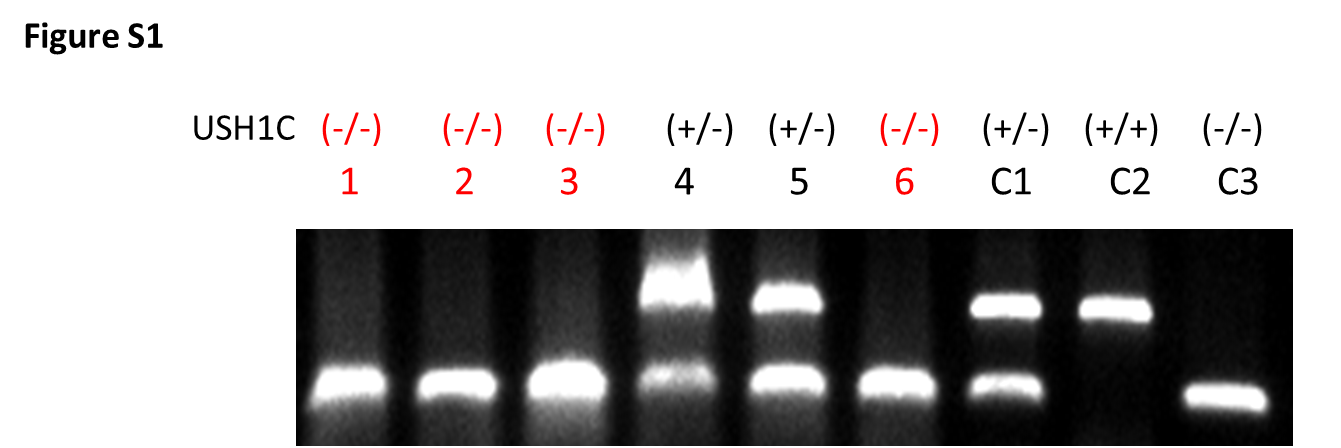


Figure S1. Representative genotyping results of USH1C mice. The mice #1, 2, 3, and 6 are identified as Ush1c KO, while #4, 5 are Ush1c (+/-). C1, C2, C3 are genotyping control with heterozygous, wild type and knockout, respectively

- **Supplemental Videos 1 & 2**: Representative videos of the swim test in GT-treated mice with the AAV2/Anc80L65.harmonin-b1 virus. Both good (V1) and poor (V2) swimming abilities are demonstrated. The untreated Ush1c KO control mice exhibit similar swimming difficulties to those shown in the poor swimming example.
- V1_KO_GT_good_Swimmer
-
- V1_KO_GT_good_Swimmer
-
- • **Supplemental Videos 3, 4, & 5**: Representative videos showing circling behavior in untreated Ush1c KO mice (V3), Ush1c KO mice treated with HarmB1 and exhibiting good swimming with no circling (V4), and those with poor swimming displaying partial circling (V5).
- V3_KO-circling
-
- V4_KO-GT-NO_Circling
-
- V5_KO_GT-partial_circling
-
- **Supplemental Table1 (Table_S1),**
